# On the 'Where' and 'When' of Eye Guidance in Real-World Scenes

**DOI:** 10.16910/jemr.12.7.6

**Published:** 2019-11-25

**Authors:** Antje Nuthmann

**Affiliations:** Perception and Cognition (Allgemeine Psychologie II) University of Kiel

**Keywords:** eye guidance, real-world scenes

## Abstract

Keynote at the 20th European Conference on Eye Movement Research (ECEM) in Alicante, 22.8.2019

**Video stream:** https://vimeo.com/361729502

